# Identification of Genome-Scale Metabolic Network Models Using Experimentally Measured Flux Profiles

**DOI:** 10.1371/journal.pcbi.0020072

**Published:** 2006-07-07

**Authors:** Markus J Herrgård, Stephen S Fong, Bernhard Ø Palsson

**Affiliations:** Department of Bioengineering, University of California San Diego, La Jolla, California, United States of America; Memorial Sloan Kettering Cancer Center, United States of America

## Abstract

Genome-scale metabolic network models can be reconstructed for well-characterized organisms using genomic annotation and literature information. However, there are many instances in which model predictions of metabolic fluxes are not entirely consistent with experimental data, indicating that the reactions in the model do not match the active reactions in the in vivo system. We introduce a method for determining the active reactions in a genome-scale metabolic network based on a limited number of experimentally measured fluxes. This method, called optimal metabolic network identification (OMNI), allows efficient identification of the set of reactions that results in the best agreement between in silico predicted and experimentally measured flux distributions. We applied the method to intracellular flux data for evolved Escherichia coli mutant strains with lower than predicted growth rates in order to identify reactions that act as flux bottlenecks in these strains. The expression of the genes corresponding to these bottleneck reactions was often found to be downregulated in the evolved strains relative to the wild-type strain. We also demonstrate the ability of the OMNI method to diagnose problems in E. coli strains engineered for metabolite overproduction that have not reached their predicted production potential. The OMNI method applied to flux data for evolved strains can be used to provide insights into mechanisms that limit the ability of microbial strains to evolve towards their predicted optimal growth phenotypes. When applied to industrial production strains, the OMNI method can also be used to suggest metabolic engineering strategies to improve byproduct secretion. In addition to these applications, the method should prove to be useful in general for reconstructing metabolic networks of ill-characterized microbial organisms based on limited amounts of experimental data.

## Synopsis

One of the major uses of in silico models in biology is to identify discrepancies between model predictions and experimental data and use these discrepancies to drive discovery of novel biological mechanisms. However, models only allow for identification of the discrepancies; they do not necessarily provide any assistance in discovering what are the missing or incorrect functionalities in the model that cause these discrepancies. Herrgård et al. describe a new in silico method, optimal metabolic network identification, or OMNI, that performs this discovery process in an efficient and systematic manner for genome-scale metabolic networks. Given a preliminary metabolic network model and experimentally determined metabolic flux data, OMNI finds the changes that need to be made to the model so that its predictions match the experimental data as well as possible. Herrgård et al. apply the method to identify metabolic bottlenecks in experimentally evolved Escherichia coli strains and to diagnose problems in strains designed through metabolic engineering strategies to overproduce specific desirable byproducts. The OMNI method can also be adapted to number of other settings, including identification of novel biochemical pathways in ill-characterized organisms based on limited amounts of experimental data.

## Introduction

Constraint-based models [1] have been successfully used to describe the steady-state functionality of genome-scale metabolic networks in a variety of microbial organism as well as specific mammalian cell types and organelles [2–4]. These models represent the metabolic network through a series of physico-chemical constraints including stoichiometric network connectivity that delineate the space of allowed metabolic fluxes in an organism. In addition to defining the space of allowed flux distributions, constraint-based models can be used to obtain a particular flux distribution by finding the optimal distribution given a particular objective function (e.g., growth or ATP production) using flux balance analysis (FBA) [5,6]. These predicted flux distributions can then be compared to experimental measurements in order to further refine the model. For example, in [3] a genome-scale metabolic model for yeast was used to predict growth phenotypes of gene deletion strains, and mispredictions were used to guide the iterative refinement of the model. However, the approach used in [3] required careful manual evaluation of the mispredictions, and in many cases clear reasons for incorrect predictions could not be identified.

Compared to other types of modeling approaches, such as kinetic models [7], constraint-based models have the advantage of having very few parameters that need to be determined from experimental data. Due to the small numbers of parameters required, genome-scale models of metabolic networks have been built using primarily information in the databases and literature without the direct use of experimental data. There are, however, situations where they may be gaps in our understanding of the metabolic network structure in a particular organism. For less well-characterized organisms there may be only limited direct experimental evidence to determine which reactions should be present in the metabolic network. Even for well-studied organisms such as Escherichia coli or yeast there may be uncertainties about specific cofactors or reaction mechanisms used in a particular reaction in a specific organism. Finally, there may be regulatory effects that prohibit the use of all the possible metabolic reactions simultaneously under any particular growth condition.

In all the cases described above one would like to identify the correct active reactions to be included in the model from a larger set of possible enzymatic reactions based on comparison between model predictions and experimental data. The experimental data types that are of particular interest for the present application are growth rates, substrate uptake, and byproduct secretion rates, as well as intracellular flux distributions measured under defined conditions [8–10]. Previously it has been found that in some cases, such as E. coli grown on acetate [11] or yeast grown on glucose [12], the FBA predictions are consistent with experimentally measured growth rate and byproduct secretion data. In other cases such as growth of E. coli on glycerol, the FBA predictions were found to be comparable with experimental data only after the strains had been adapted to the growth environment by evolving them experimentally for hundreds of generations [13]. However, it is also possible that the FBA predictions remain inconsistent with experimental data even after adaptation to a particular growth environment as is the case for some metabolic gene knock-out strains of E. coli [14].

In cases where major discrepancies between model predictions and experimental data exist even after adaptation to a particular growth environment, methods need to be developed to systematically find minimal modifications to the metabolic network structure that improve model predictions. The changes identified in this fashion can then be validated by direct experimental techniques targeting specific novel mechanisms suggested by the computational analysis. Efficient methods for identifying parameters in small-scale kinetic models of biochemical networks have been developed [15–17]. Methods have also been developed for constraint-based models to identify objective functions that are consistent with experimentally measured metabolic flux data [18,19]. However, approaches for identifying reaction complements of genome-scale metabolic models have not been presented before.

**Figure 1:**
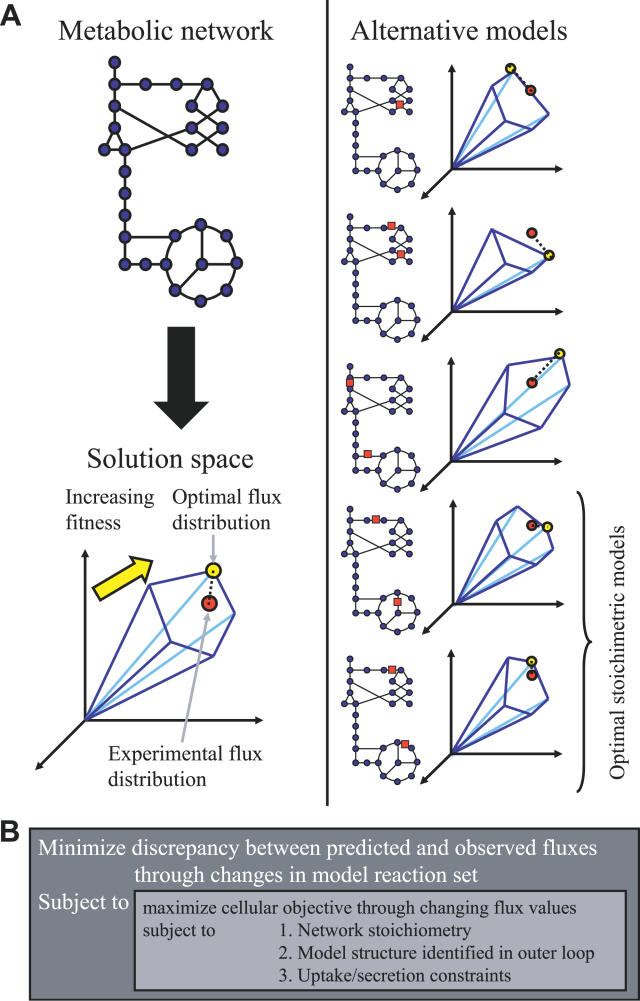
Bilevel Approach to OMNI (A) Schematic illustration of the optimal metabolic network identification approach. Changes in the model reaction set lead to changes in the FBA-predicted optimal flux distribution (yellow) that can be compared to the experimental fluxes (red). (B) Bilevel optimization scheme for optimal metabolic network identification.

In this paper the development and application of computational methods for optimal metabolic network identification (OMNI) based on in vivo measured growth rate, exchange flux (substrate uptake and byproduct secretion rate), and intracellular flux data is described. The model identification method uses a bilevel mixed-integer optimization strategy introduced in [20] to identify the optimal network structure given one or more sets of experimentally determined metabolic flux data. It is assumed that most of the reactions in the network are active, and only a small fraction of the reactions in the model can be either excluded from or included in the list of active reactions. The task is then to find which reactions need to be included in the model or removed from the model to make the model predictions agree with the experimental data as closely as possible (see Figure 1 for a schematic illustration of this process). In the present paper we focus exclusively on applying OMNI to experimentally evolved strains, but the method is not limited to this specific application. Some of the potential applications of the OMNI method are listed in Table 1.

**Table 1:**
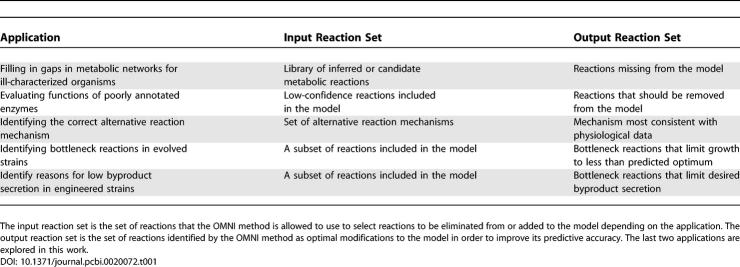
Potential Applications of the OMNI Method

The OMNI method is illustrated by using it to identify potential flux bottlenecks based on experimental data for five different E. coli knock-out strains evolved for 45–50 d on glucose [14,21]. For each of these strains the original genome-scale metabolic model [2] over predicts the growth rate compared with the experimental data. The OMNI approach allows identifying particular bottleneck reactions in the model whose removal improves the agreement between the model predictions and experimental data. In order to provide further support for the flux bottleneck role of the reactions identified by OMNI, we also analyze gene expression data for the evolved strains to find if the genes corresponding to the bottleneck reactions are downregulated in these strains. In the second study the OMNI approach is used as a diagnostic tool to identify flux bottlenecks in an E. coli strain designed through metabolic engineering to overproduce lactate [20]. We used the OMNI method to identify potential in vivo flux bottlenecks that could explain the lower-than-predicted performance of the strain. This application illustrates the utility of the OMNI method in assisting strain development and identifying potential novel metabolic engineering strategies.

## Results

### Evolved Metabolic Gene Knockout Strains

FBA applied to genome-scale metabolic network models has been shown to correctly predict the physiological end points of laboratory evolution of wild-type [13] and metabolic gene knockout strains of E. coli [21]. However, for a small fraction of the knockout strains, the model significantly overpredicts growth rates compared to data obtained for the endpoint strains after 45–50 d of experimental evolution adaptation to glucose-minimal medium [21]. In these cases one would like to characterize the behavior of the strains and to identify reasons for discrepancies between model predictions and experimental data. Previously, metabolic flux and gene expression profiling have been applied to characterize the physiological and expression states of evolved strains with behavior inconsistent with model predictions [14]. In particular, two independently evolved endpoint strains for each of the original deletion strains harboring triose phosphate isomerase *(tpi),* phosphoenolpyruvate carboxylase *(ppc),* and phosphoglucose isomerase *(pgi)* gene deletions were characterized. The growth rates of these strains were overpredicted by the E. coli iJR904 genome-scale metabolic model [2] by an average of 22% compared to the endpoint strains obtained by experimental evolution. The physiological characteristics of the parental deletion strains and the evolved strains are described in Table 2.

**Table 2:**
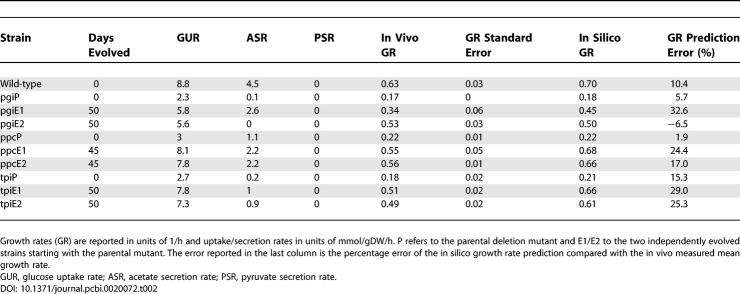
Experimentally Measured Physiological Parameters of Unevolved and Evolved Knockout Strains and Predicted Growth Rates from E. coli iJR904 Model

In order to discover potential causes for the growth rate prediction discrepancies we applied the OMNI method to each of the endpoint strains. We did not include the pgiE2 strain in the analysis, since the model did not overpredict the growth rate of this strain, and hence it was not expected that the OMNI method could improve the model predictions. Because all the discrepancies between experimental data and in silico predictions were overpredictions, we assumed that these were caused by reactions in the model that for some reason could not operate at full capacity in the evolved strains. In order to apply the OMNI method to the evolved deletion strain data, we deleted in silico the reactions corresponding to the genes deleted in each parental strain (*pgi*Δ, *ppc*Δ, and *tpi*Δ) and set the uptake/secretion rates to the mean values listed in Table 2. The set of measured target fluxes used in the objective function of OMNI consisted of the experimentally measured growth rate and 23 intracellular fluxes reported in [14]. We applied the OMNI method to each of the five evolved strains with one to four reaction deletions allowed. Increasing the number of allowed reaction deletions beyond four did not result in significant improvements in model predictions.

**Figure 2:**
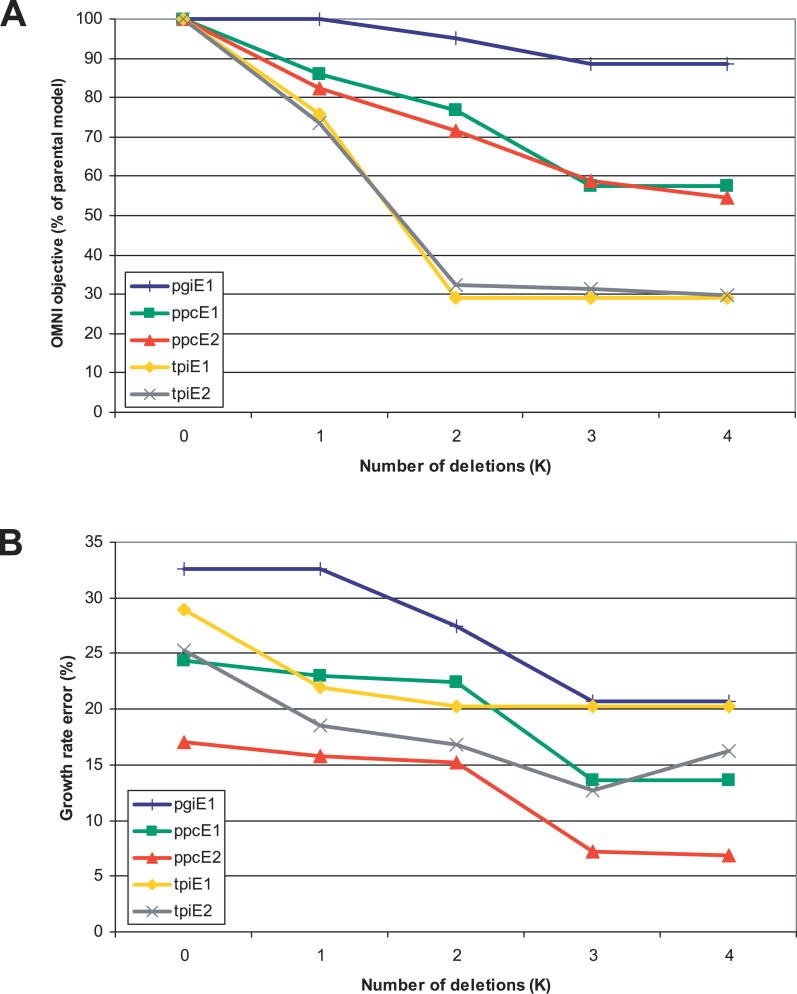
The Dependence of the Model Prediction Errors on the Number of Reactions Deleted from the Parental Strain Model (A) The overall combined intracellular and growth rate prediction error as measured by the percentage of the OMNI objective value for the optimal modified model compared with the objective value for the parental strain model. (B) The growth rate prediction error (percentage of the experimental growth rate) for the optimal modified model identified by the OMNI method.

The results from this study are summarized in Table 3, which indicates the optimal one-to-four-reaction bottleneck sets for each strain that was identified by the OMNI method. This table also shows the values of the OMNI objective function measuring the overall error in all flux predictions and the error in growth rate prediction for each of the modified models identified by OMNI. All the alternative optimal reaction bottleneck sets with the same OMNI objective value as well as all other suboptimal bottleneck sets identified in the calculations are listed in Table S1. For all five strains studied, OMNI was capable of identifying a modified model that predicts both growth rates and intracellular fluxes significantly better than the parental strain model. Increasing the number of reaction deletions also improved the overall agreement between model predictions and experimental data for all strains (Figure 2A). Across all five strains the average improvement in the OMNI objective function in the best possible modified model identified by the method compared with the parental strain model was 48%. Increasing the number of modifications generally decreased the growth rate overprediction compared with the parental strain model (Figure 2B), but for the tpiE2 strain there was a trade-off between accurate prediction of intracellular fluxes and growth rate.

**Table 3:**
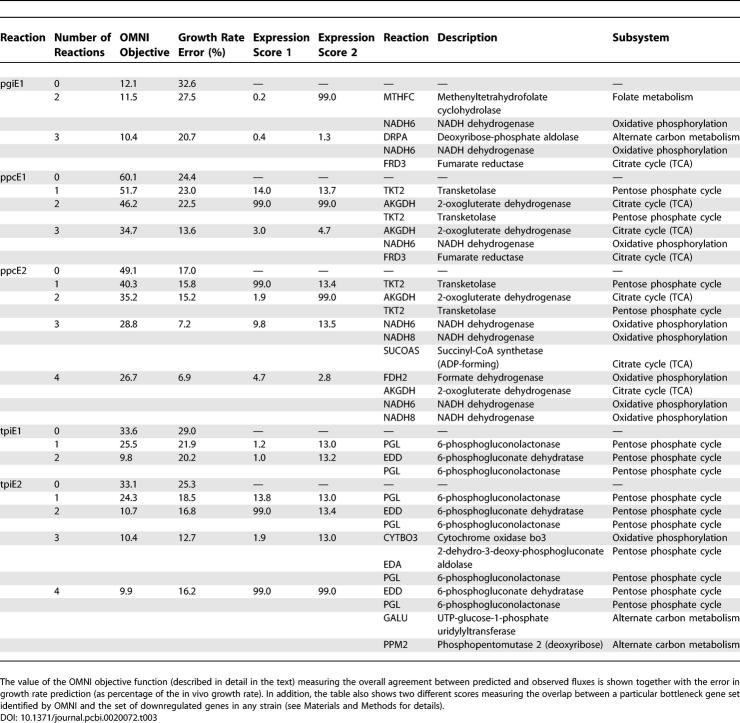
Reaction Bottlenecks for the pgiE1, ppcE1, ppcE2, tpiE1, and tpiE2 Strains Identified Using the OMNI Approach

**Figure 3:**
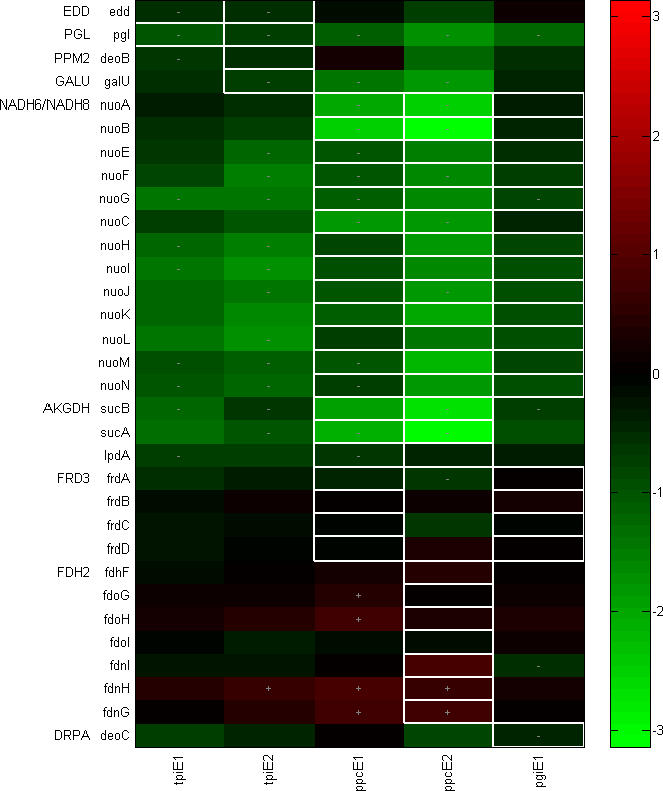
Correspondence between Bottleneck Reactions Identified by the OMNI Approach and Gene Expression Changes in the Evolved Knockout Strains Relative to the Wild-Type E. coli Strain Expression changes, reported as log_2_ ratios and statistically significant expression changes (see Materials and Methods for details) in each evolved strain, are shown by +/− signs depending on the direction of the change. The reaction names are listed in the first column and the corresponding genes are listed in the second column. The genes corresponding to the best combination of reaction bottlenecks of up to three reactions identified by the OMNI approach (Table 3) for each strain are shown by white boxes.

We also investigated the correspondence between the bottleneck reactions identified by the OMNI method and the experimentally determined gene expression changes in the evolved strains compared with the wild-type E. coli strain [14]. For this purpose we identified the genes associated with each of the bottleneck reaction sets in the iJR904 model. The statistical significance of the overlap between the sets with downregulated genes in each strain and the bottleneck gene sets was quantified by the significance-based (expression score 1) and sign-based (expression score 2) scores as described in Materials and Methods. The expression scores reported in Table 3 indicate that the gene sets corresponding to the bottleneck reactions were indeed enriched in genes that were downregulated at the expression level. However, the downregulation was not always at a statistically significant level (pgiE1 and tpiE1 strains). The gene expression changes corresponding to the optimal bottleneck gene sets in all the five strains studied are summarized in Figure 3. The gene expression changes in all strains were quite similar despite their different physiological characteristics, and despite the significant differences in the bottleneck reaction sets identified by OMNI.

### Lactate-Overproducing *ptaA-pfk* Strains

The OptKnock in silico strain design methods suggests gene deletion strategies that link overproduction of a desired byproduct to biomass production through stoichiometric coupling [20]. The idea is that the designed strains could then be evolved in laboratory conditions towards higher growth rates, and at the same time, they could achieve higher byproduct secretion rates. This approach was applied to the iJR904 model to design two- and four-gene deletion strains of E. coli that overproduce lactic acid [20]. Previously, three of these designs were implemented in vivo, and three independent experimental evolutions in anaerobic glucose minimal media conditions of up to 60 d were performed starting with each parental strain [22]. Strains derived from two of the three designs achieved the predicted optimal lactate secretion and growth rates after 60 d. However, after 60 d of evolution, strains derived from one of the parental deletion strains (*pfkA-pta* double deletion) had growth rates that were 18%–32% lower than what was predicted by FBA. This strain actually evolved to the predicted optimal phenotype after 20 d, but further experimental evolution resulted in a suboptimal phenotype.

We applied the OMNI method as a diagnostic tool to identify reasons for the suboptimal growth of the evolved *pfkA-pta* strains. The physiological data for the three independently evolved strains that were used as an input to the OMNI approach are listed in Table 4. Glucose uptake and lactate secretion were used as constraints in the OMNI approach, and the objective consisted of the growth rate and acetate/ethanol secretion rates. No intracellular flux data was used in this application of the OMNI method. OMNI identified the same one- and two-reaction bottleneck sets in each of the three strains. Further reaction deletions in the model reduced the objective function somewhat, but resulted in higher growth rate prediction errors, and hence were excluded from further analysis (all solutions are listed in Table S2). The reaction bottlenecks identified in all the strains were either PDH (pyruvate dehydrogenase) alone or PDH together with ATPS4r (ATP synthase). The OMNI objective values and growth rate prediction errors were both reduced significantly for the modified models compared with the parental strain model (Table 5). In particular, the error in growth rate prediction is reduced to less than 10% of the experimental growth rate for all three independently evolved strains. The three evolved strains were also subjected to gene expression profiling using oligonucleotide arrays, and significantly downregulated genes in each of the evolved *pfkA-pta* strains compared with the wild-type E. coli were identified (Materials and Methods). The bottleneck-associated genes were significantly enriched in downregulated genes in all three strains (Table 5).

**Table 4:**
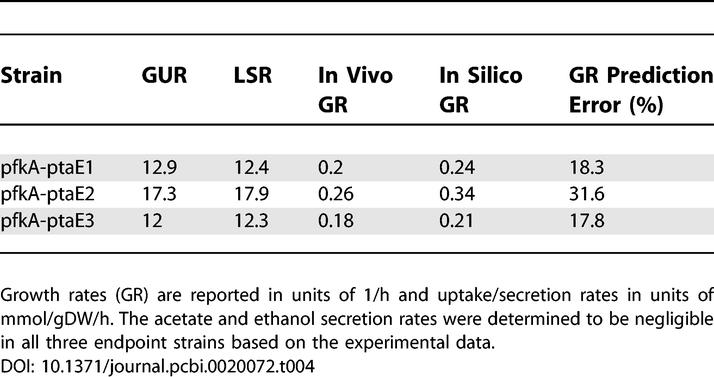
Physiological Parameters of the 60-d Evolved *pfkA-pta* Strains under Anaerobic Conditions

**Table 5:**
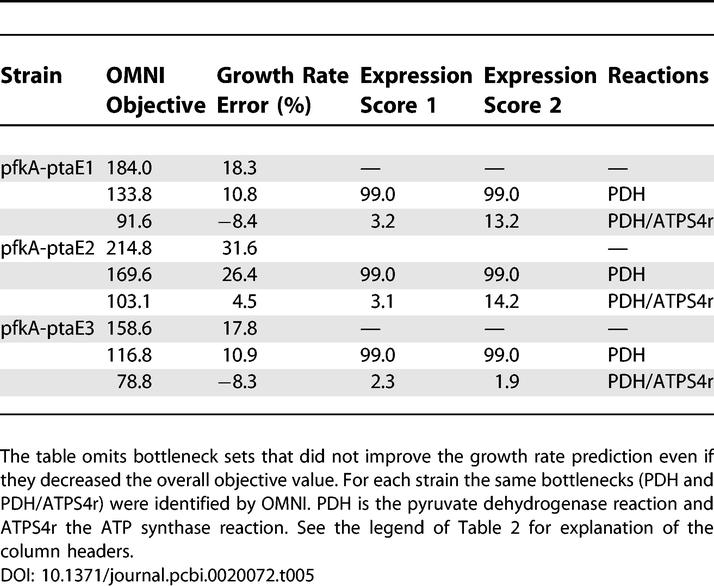
Reaction Bottlenecks Identified Using the OMNI Approach for the Evolved Lactate-Overproducing Strains pfkA-ptaE1–3

## Discussion

We have introduced a new approach, OMNI, for identifying changes in the reaction complement of a genome-scale metabolic model that are needed to minimize the discrepancy between model predictions of optimal flux distributions and experimentally measured flux data. Based on earlier experimental work it is known that the optimal predictions should be quantitatively comparable to measured uptake, secretion, and growth rates for microbial strains adapted to specific growth conditions through experimental evolution. The application of the OMNI approach explored in this paper is using it as a tool to identify potential sources of model mispredictions when exchange or intracellular flux data for experimentally evolved strains is available.

When applied to physiological data from five experimentally evolved E. coli knockout strains, the OMNI method identified potential bottleneck reactions in the iJR904 model; removing those reactions from the model reduced the discrepancy between experimentally measured and predicted fluxes (Figure 2). The deletion of at least two reactions was required in order to improve agreement between model predictions and experimental data significantly. This result indicates that a full enumeration of all possible reaction deletions would be highly inefficient compared with the bilevel optimization approach used in the OMNI method. Typically, the bottlenecks identified for the two independently evolved strains, starting with the same parental strain, were exactly the same or at least in the same metabolic subsystem. Even in cases where the optimal bottleneck sets for the two independently evolved strains were different, such as ppcE1 and ppcE2, there were slightly suboptimal solutions for each strain that were more similar with each other than the optimal solutions (Table S1).

The comparison between gene expression changes in evolved strains and the bottlenecks identified by the OMNI approach showed that in many cases the genes corresponding to the bottleneck reactions were downregulated in the evolved strains compared with the wild-type strain. Even when only genes that were statistically significantly downregulated (false discovery rate of 5%) in the evolved strain were considered, most of the gene sets corresponding to the bottleneck reactions reported in Table 3 were significantly enriched in downregulated genes. Only in the pgiE1 and tpiE1 strains did the genes corresponding to most optimal bottleneck sets fail to show a significant degree of downregulation. The actual expression profiling data shown in Figure 3 indicates a source of potential discrepancies between gene expression changes and bottleneck reactions identified using OMNI. In many cases, genes corresponding to a reaction identified as a bottleneck by OMNI act as isozymes or are part of a complex catalyzing the same reaction. In these cases only one of the isozymes or complex members may be downregulated, and this downregulation may be sufficient to restrict the flux through the corresponding reaction significantly.

The combination of the OMNI approach applied to experimentally measured flux data together with gene expression profiling data allows us to identify potential regulatory constraints that may limit the ability of strains to evolve to achieve predicted optimal growth phenotypes. For example, in the case of the tpiE1 and tpiE2 strains, the key bottlenecks identified by OMNI were in the pentose phosphate pathway (PGL/G6PDH2r and EDA/EDD), and the expression data strongly supported the downregulation of this pathway in the evolved strains. It is not clear why the expression of the genes in the pentose phosphate pathway is lower in the evolved strain than in the wild-type strain, since allowing maximal possible flux through this pathway should be advantageous for the cell based on the model predictions. One possibility is that the cells are trying to reduce NADPH production by this pathway in order to compensate for the partial loss of ability to consume this cofactor by reactions linked to the deleted *tpi* gene reaction.

As described above the *pfkA-pta* deletion strain studied here was designed using the OptKnock computational strain design to maximize the production of lactate while still maintaining reasonable growth rate [20]. Here, we used the OMNI approach, which is a modification of the same bilevel optimization procedure that OptKnock uses to diagnose why the designed strain did not perform as well as the model predicted. In particular, the results obtained here suggest that the primary bottleneck in the evolved *pfkA-pta* strains would be the PDH reaction. This conclusion is also supported by the expression profiling data that for all three independently evolved strains shows significant downregulation of the subunits of the PDH complex (Table 5). It is possible that overexpressing components of the PDH complex in the evolved *pfkA-pta* strain might remove the growth rate limitation, but since this also would potentially result in higher ethanol production, more complex strain engineering might be required to improve the growth rate of the *pfkA-pta* strain. The OMNI approach proposed in this work would then not only be useful for model building, but could also be used to aid in developing improved strains for metabolic engineering applications.

The application of the OMNI method to evolved E. coli strains demonstrated only one potential use of the method. The use of the OMNI method is not limited to experimentally evolved strains, as variants of FBA, such as minimization of metabolic adjustment (MOMA) [23], have been shown to allow accurate prediction of metabolic phenotypes for nonevolved metabolic gene deletion strains. The inner FBA problem in the bilevel optimization procedure that is used in the OMNI approach can be replaced by a variant of the MOMA method [20]. With this modification the OMNI method should also prove to be useful in characterizing sources of mispredictions for nonevolved strains. For ill-characterized organisms the combined OMNI/MOMA method would allow refinement of the network structures of specific subsystems based solely on systems level data such as exchange fluxes. For this type of application it may be necessary to use flux data measured in multiple different media conditions that require the use of different reaction sets. The inner problem of the OMNI approach can include multiple independent FBA or MOMA problems representing different media conditions or gene deletion strains so that the OMNI approach can be adapted to simultaneously use multiple flux datasets.

Using multiple datasets generated under different conditions would also avoid potential issues with over fitting the model to only one flux profile. In particular, some of the flux datasets could be used to train the model using the OMNI approach while the predictive ability of the modified models identified by OMNI could be tested against a set of independent flux datasets. In the applications to evolved strains discussed in this paper it would be possible to apply the OMNI method to multiple flux profiles from the independently evolved endpoint strains for each parental strain (e.g., ppcE1 and ppcE2). However, these strains are not necessarily genetically identical, and hence it may not be appropriate to try to identify common flux bottleneck sets for both strains.

### Conclusions

We have developed a method, OMNI, to identify potential changes to genome-scale metabolic models based on comparing model predictions of fluxes to experimental measurements. The method uses mixed-integer linear programming to efficiently search through the space of potential metabolic model structures. We applied the method to intracellular flux data for five experimentally evolved E. coli strains to identify why the model predictions for growth rates of these strains were an average of 22% higher than the actual experimental measurements. The OMNI method identified specific bottleneck reactions in the iJR904 metabolic model that, when removed from the model, would reduce the discrepancies between predicted and observed fluxes significantly. Genes corresponding to these reactions were often found to be downregulated in the evolved strains relative to the wild-type strain. We also applied OMNI to strains created by metabolic engineering approaches and experimentally evolved to optimize their metabolite production potential [20,22,24,25]. In this application, OMNI could be used to identify sources of lower-than-predicted secretion of desired byproducts and to suggest potential ways to improve the strain designs. In summary, the OMNI method provides an efficient and flexible way to study and refine genome-scale metabolic network reconstructions using limited amounts of experimental data.

## Materials and Methods

### FBA.

The FBA approach applied to genome-scale constraint-based metabolic models can be used to make predictions of flux distributions based on linear optimization [5,6]. In mathematical terms, a prediction for the metabolic flux distribution vector *v* is obtained as the solution of


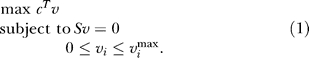


All the definitions of the variables used in this section are summarized in Table 6. The objective coefficients are usually derived from the cellular biomass composition so that the maximum value of the objective function corresponds to the predicted optimal growth rate given the network stoichiometry and media composition represented by upper bounds on exchange reactions. The linear nature of these problems allows developing methods for model structure identification that are significantly different from methods that would have to be used in modeling frameworks based on systems of ordinary differential equations [17,26].

**Table 6:**
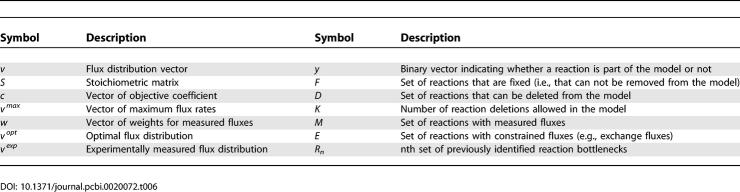
Descriptions of Variable Names Used in Equations 1–4

### OMNI.

The problem of finding the best possible metabolic reaction set to match model predictions with experimental flux data can be formulated as a bilevel optimization problem, as shown in Figure 2. The outer optimization problem searches through a set of reactions to include in the model, and the inner optimization problem produces a flux distribution as a solution to a FBA problem (Equation 1) given a particular model structure. Mathematically this problem can be expressed as (see Table 6 for definitions of the variables used):


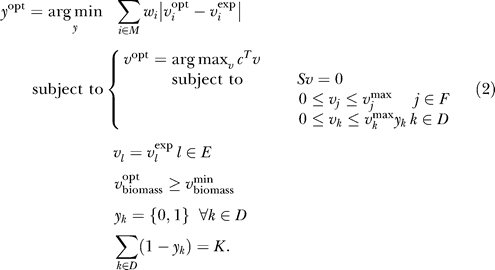


The nonzero elements of the vector *y* indicate the reactions that are included in the model and the zero elements the reactions that are excluded from the model. The bilevel optimization formulation is similar to the one used in the OptKnock computational strain design approach [20]. The major difference is the objective function for the outer problem, where OptKnock only uses a single flux instead of a distance between predicted and experimentally determined flux profiles. In addition, the set of constraints *E* includes measured exchange fluxes that are not used in the OptKnock approach.

In the applications described in this paper the measured exchange fluxes (set *E*) are not used as part of the objective except in the case of the *pfkA-pta* strains; instead, the exchange fluxes are set explicitly to their measured values. This is done because at least one of the fluxes has to be bounded for the inner optimization problem to have a bounded solution. By fixing the exchange fluxes we ensure that the flux distributions satisfy at least these constraints and reduce the problem to finding a model that minimizes the discrepancy between the remaining experimentally measured and predicted fluxes. In general, it is possible to move fluxes between sets *E* (constraints) and *M* (objective function). The fluxes in set *E* are assumed to be more accurately measured than those in set *M* so that in most applications intracellular fluxes would be included in set *M* and exchange fluxes in set *E*.

The reactions in the model are assumed to be partitioned into a set of fixed reactions that cannot be removed from the model *(F),* and a set of reactions that can be deleted *(D).* For example, the set *F* could be the set of reactions included in the model with high confidence and *D* could be a set of potential reactions inferred (e.g., by sequence homology only or all known biochemical reactions not included in set *F* from a large database such as the Kyoto Encyclopedia of Genes and Genomes [KEGG]) [25,27]. The stoichiometric matrix *S* contains the stoichiometric coefficients for all reactions in both sets *D* and *F,* but only parts of this matrix would be used as constraints depending on the nonzero elements in *y.* In the inner optimization problem a standard FBA problem is solved for a particular metabolic network structure defined by the set of binary variables *y_k_.* The outer problem, on the other hand, treats the elements of the vector *y* as variables in its own optimization problem that aims to minimize the discrepancy between observed *(v^exp^)* and predicted optimal flux *(v^opt^)* distributions. The outer problem will systematically search over the space of possible integer vectors *y* to find the one that minimized the discrepancy, and hence discover the best possible metabolic model given the experimental flux data.

Solving the bilevel optimization problem directly using combinatorial approaches would be time consuming due to the large number of possible combinations of reactions that could be added to the model or removed from the model. Fortunately, the linear programming nature of the inner problem allows formulating the overall problem as a single mixed-integer linear program (MILP) following the approach developed in [20]. This formulation is based on duality theory and amounts to converting the inner linear program to a larger set of equalities and inequalities that every optimal solution to the linear program has to satisfy [28]. By applying the primal–dual approach to the bilevel optimization problem (Figure 2), the original bilevel problem can be written as a single large optimization problem as shown in [20]. The conversion of the inner problem from an optimization problem to a set constraint also bypasses the issue with alternative optimal solutions to the inner FBA problem [29]. The OMNI method will always evaluate the distance between predicted and experimentally determined flux vectors (i.e., the OMNI objective function) based on the alternative optimal flux vector that is closest to the experimental one.

The objective function in Equation 2 can be expressed in an equivalent form that only involves linear terms and constrains as:


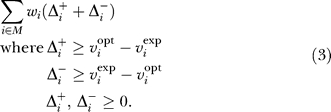


Using this form of the objective function converts the overall problem (Equation 2) into a single large MILP. After identifying one potential set of model changes we also identified other reaction deletions that would result in the same prediction by repeatedly running the OMNI algorithm and requiring it to find a solution that is different from all of the previously found ones. This requirement can be implemented by adding the following additional constraints to problem (Equation 2):


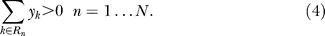


Here *N* is the total number of previously obtained solutions. The OMNI algorithm was run with these additional constraints until no further solutions that would reduce the error between the predicted and observed flux distribution could be found.

This MILP can be solved in most cases to optimality in a reasonable time (a few hours) using standard MILP solvers run on a single workstation. For genome-scale models the solver time can be reduced significantly by first identifying the tightest possible upper and lower bounds for each reaction using flux variability analysis [30]. For many reactions these bounds turn out to be zero under in a particular environment so that these blocked reactions and their corresponding integer variables can be removed from the model without any loss of information. In this work the CPLEX 9.0 MILP solver was used through the GAMS interface (GAMS Development Corp., Washington, D. C., United States). The Matlab scripts used to set up the OMNI optimization problems and to solve the problem using CPLEX through GAMS are provided in Dataset S1.

### E. coli model.

The E. coli iJR904 genome-scale metabolic model containing 1,075 reactions catalyzed by 904 distinct enzymes [2] was used as the base model for this study. For each particular gene deletion strain studied, the reactions catalyzed by the deleted genes where removed form the model to obtain the parental strain model. For the purposes of the OMNI approach we considered all the reactions involved in central metabolism, including oxidative phosphorylation and alternative carbon source metabolism (227 reactions total), to be potential candidate bottleneck reactions (set *D* in Equation 2). In the OMNI objective each of the discrepancies between predicted and measured fluxes was weighted in proportion to the inverse of the experimental standard deviation of the flux measurement (*w_i_* in Equation 2). Furthermore, in order to bias the search towards network structures that would result in correct growth rate predictions, the growth rate error was weighted as 80% of the overall objective, and the cumulative error over all the other fluxes in the objective function was weighted as 20% of the overall objective. We also performed the OMNI calculations with other weight values (90% and 100% growth rate weight), and these are reported in Protocol S1. The optimal bottleneck sets for the calculations where the intracellular flux data was used (80% and 90% growth rate weight) were generally consistent with each other.

### Gene expression profiling and data analysis.

The gene expression profiling for the three independently evolved *pfkA-pta* deletion strains was done as described in [14] using Affymetrix (Santa Clara, California, United States) E. coli Antisense Genome Arrays. Three biological replicates were used for each of the three evolved *pfkA-pta* deletion strains and six biological replicates for the wild-type strain all grown aerobically on glucose-minimal media. Probe intensity calculations and normalization was performed using the Robust Multichip Average (RMA) method [31] and differentially expressed genes between each evolved strain and wild-type were detected using a standard *t* test applied to log-transformed data. The *p* value cut-off for significance was determined for each strain separately using the Benjamini-Hochberg approach [32], with a false discovery rate of 5%. For each set of bottleneck reactions the corresponding genes were identified based on the gene-reaction associations in iJR904 [2]. In order to assess whether the genes in a particular bottleneck gene set were significantly enriched in the set of downregulated genes we used the hypergeometric distribution to calculate the probability *p* that the observed overlap between the gene sets occurs by random chance. The gene expression overlap scores reported in Tables 2 and 4 are then defined as −log_10_ (*p*). For the expression score 1 (significance-based score), the set of downregulated genes was determined by using the *p* value threshold determined as described above and requiring that the log_2_ ratio between mean evolved deletion strain and wild-type expression levels was negative (i.e., gene is downregulated). For the expression score 2 (sign-based score), the downregulated genes were determined based on the sign of the log_2_ ratio between the evolved knockout strain and the wild-type strain, ignoring the significance of the expression change. If the *p* value used to calculate either score was exactly equal to zero (corresponding to case where all the bottleneck genes are downregulated), the score was assigned the value 99 in Tables 2 and 4.

## Supporting Information

Dataset S1 Compressed Archive of the Matlab and GAMS Scripts Needed to Run OMNI Calculations (40 KB ZIP)

Protocol S1 Supplementary Results Document Describing Sensitivity Analysis of the Results Presented in the Paper (33 KB DOC)

Table S1 Optimal and Suboptimal Bottleneck Reaction Sets Identified for the Five Single-Deletion Strains (30 KB XLS)

Table S2 Optimal and Suboptimal Bottleneck Reaction Sets Identified for the pfkA-pta Strain (20 KB XLS)

Table S3 Gene Expression Datasets for the Three Evolved Strains Derived from the *pfkA-pta* Parent Strain (1.6 MB XLS)

### Accession Numbers

The EcoCyc (http://biocyc.org/ECOLI/NEW-IMAGE?type=GENE&object=b1851) accession numbers for the genes discussed in this paper are *tpi* (b3919), *ppc* (b3956), *pgi* (b4025), *pfkA* (b3916), *pta* (b2297), *lpdA* (b0116), *sucA* (b0726), *sucB* (b0727), *pgl* (b0767), *galU* (b1236), *fdnG* (b1474), *fdnH* (b1475), *fdnI* (b1476), *edd* (b1851), *nuoN* (b2276), *nuoM* (b2277), *nuoL* (b2278), *nuoK* (b2279), *nuoJ* (b2280), *nuoI* (b2281), *nuoH* (b2282), *nuoG* (b2283), *nuoF* (b2284), *nuoE* (b2285), *nuoC* (b2286), *nuoB* (b2287), *nuoA* (b2288), *fdoI* (b3892), *fdoH* (b3893), *fdoG* (b3894), *fdhF* (b4079), *frdD* (b4151), *frdC* (b4152), *frdB* (b4153), *frdA* (b4154), *deoC* (b4381), and *deoB* (b4383).

## Abbreviations

FBA: flux balance analysis
MILP: mixed-integer linear program
MOMA: minimization of metabolic adjustment
OMNI: optimal metabolic network identification
PDH: pyruvate dehydrogenase
*pgi*: phosphoglucose isomerase
ppc: phosphoenolpyruvate carboxylase
*tpi*: triose phosphate isomerase
