# Supplementary material for: Identification of Genome-Scale Metabolic Network Models Using Experimentally Measured Flux Profiles
Source: PLoS Comput Biol. 2006 Jul 7;2(7):e72. doi: 10.1371/journal.pcbi.0020072 (PMC1487183; doi:10.1371/journal.pcbi.0020072)
Supplement: Supplementary file 2 [file pcbi.0020072.sd002.doc]

**Protocol S1: Supplementary Results**

Markus J. Herrgård, Stephen S. Fong, and Bernhard O. Palsson

*Identification of Genome-Scale Metabolic Network Models Using Experimentally Measured Flux Profiles*

**Sensitivity analysis of OMNI results**

The objective function for the OMNI problem can be separated into growth (or biomass production) rate and intracellular flux terms

$\begin{matrix} \sum_{i\in M} w_{i}\left|v_{_{i}}^{\text{opt}}-v_{i}^{\exp}\right|=\gamma w_{biomass}\left|v_{biomass}^{opt}-v_{biomass}^{\exp}\right|+(1-\gamma)\sum_{i\in MI} w_{i}\left|v_{_{i}}^{\text{opt}}-v_{i}^{\exp}\right| \\ \text{ } \end{matrix}$,

where *MI* is the set of measured intracellular fluxes. The weights *w_i_* for each measured flux are in proportion to the inverse of the experimental standard deviation for the corresponding flux. The additional factor γ is used to allow biasing the OMNI search towards solutions that improve growth rate predictions. The results presented in the paper were obtained with γ=0.8 corresponding to weighing the growth rate discrepancy as 80% of the overall objective and all the internal fluxes as 20% of the overall objective. In order to investigate the effect of the γ parameter on the OMNI results we performed OMNI optimization with two additional values of γ (0.9 and 1.0). These results, presented in Table S.3, show that except for the pgiE1 strain the results for the 80% and 90% growth rate bias are very similar. However, in general when the intracellular flux measurements were ignored entirely (100% case), the OMNI bottleneck sets changed quite significantly.

**Table S.1 Bottleneck reactions identified by OMNI with different weights used in the objective function.** Results are shown for cases where the growth rate is weighted as 80%, 90% or 100% of the total objective. Correspondingly the intracellular fluxes are weighted as 20%, 10% and 0% of the total objective in each case.

**Using part of the flux data to test model predictions**

We also investigated the possibility of using an approach where only part of the flux data for each strain is used to find an optimal model with OMNI and the ability of this model to predict remaining fluxes is determined separately. For this purpose we first identified optimal bottleneck sets for each strain using only the growth rate in the objective function ignoring intracellular fluxes (this corresponds to the 100% case reported in Table S.1). Then we restricted the fluxes through the identified bottleneck reactions to zero and computed the discrepancy between predicted and experimentally determined intracellular fluxes:

$$\begin{matrix} \text{Discrepancy}=\sum_{i\in MI} w_{i}\left|v_{_{i}}^{\text{opt}}-v_{i}^{\exp}\right| \\ \text{ } \end{matrix}$$

This calculation was performed using the OMNI algorithm with *K* = 0 so that the alternative optimal solution closest to the experimental flux distribution is used to calculated the flux discrepancy We also evaluated the same discrepancy for the parental strain model with no bottleneck reactions and this value was used as baseline value for each strain. The results of the calculations described above (Table S.2) indicate that for all but four bottleneck sets the model predicts intracellular fluxes worse than the parental model. This indicates that if the model is trained using the growth rate data only it will usually fail to predict the correct intracellular flux patterns.

An alternative approach to partitioning the data would be to use a subset of the measured intracellular fluxes in training the model in addition to the growth rate data. However, the intracellular fluxes in each flux distribution are dependent on each other due to the process used to estimate them from the primary experimental measurements on mass isotope distributions of proteinogenic amino acids [1]. For this reason there does not appear to be a reasonable way to split any one flux profile into training and testing sets. As described in the main paper the most appropriate approach would be to have multiple independent flux profiles for each strain measured under different cellular conditions and use some of the flux profiles for training metabolic network models and others to test the trained models. However, we do not currently have such large scale flux data sets for the strains under consideration here.

**Table S.2. Discrepancies between predicted and measured intracellular flux profiles for models trained using only growth rate data.**

1. Fong SS, Nanchen A, Palsson BO, Sauer U (2006) Latent pathway activation and increased pathway capacity enabl*e Escherichia co*li adaptation to loss of key metabolic enzymes. J Biol Chem 281:8024-33.
